# Coverage and determinants of childhood vaccination during the COVID-19 pandemic in Fortaleza, Northeastern Brazil: a longitudinal analysis

**DOI:** 10.1590/0102-311XEN074723

**Published:** 2024-02-02

**Authors:** David Augusto Batista Sá Araújo, Luciano Lima Correia, Pedro Lucas Grangeiro de Sá Barreto Lima, Sophia Costa Vasconcelos, Simone Farías-Antúnez, Yuri Valentim Carneiro Gomes, Denise Lima Nogueira, Márcia C. Castro, Marcia Maria Tavares Machado

**Affiliations:** 1 Faculdade de Medicina, Universidade Federal do Ceará, Fortaleza, Brasil.; 2 Departamento de Ciencias da Saúde, Universidade Federal de Santa Catarina, Araranguá, Brasil.; 3 Faculdade Luciano Feijão, Sobral, Brasil.; 4 Harvard University, Boston, U.S.A.

**Keywords:** Vaccines, COVID-19, Vaccination Coverage, Longitudinal Studies, Vacinas, COVID-19, Cobertura Vacinal, Estudos Longitudinais, Vacunas, COVID-19, Cobertura de Vacunación, Estudios Longitudinales

## Abstract

Brazil has seen a decrease in vaccination coverage since 2016. This study analyzes the immunization status of children born during the COVID-19 pandemic in Fortaleza, Northeastern Brazil. This is a longitudinal analysis that included vaccination data of 313 children aged 12 and 18 months. Vaccination cards were checked for dose application considering the schedule of immunization recommended by the Brazilian Ministry of Health. Factors associated with no retention of vaccination cards and incomplete immunization by 18 months were identified by Tobit regression analysis. About 73% of mothers presented their child’s vaccination card. Non-availability of vaccination cards was associated with maternal age < 25 years and mothers with paid jobs. Only 33% and 45% of the children aged 12 and 18 months had all vaccines up to date, respectively. For 3-dose vaccines, the delay rate was around 10% for the first dose application, but 40% for the third dose. Despite delays, most children with available vaccine cards had coverage above 90% by 18 months of age. Adjusted factors associated with incomplete vaccination included living in a household with more than one child (p = 0.010) and monthly income of less than one minimum wage (p = 0.006). Therefore, delays in child vaccine application were high during the COVID-19 pandemic but a considerable uptake by 18 months of age was found. Poorer families with more than one child were particularly at risk of not fully immunizing their children and should be the target of public policies.

## Resumo

O Brasil registra uma diminuição na cobertura vacinal desde 2016. Este estudo analisa a situação vacinal de crianças nascidas durante a pandemia de COVID-19 em Fortaleza, Nordeste do Brasil. Uma análise longitudinal incluiu 313 crianças com informações aos 12 e 18 meses de idade. A aplicação das doses foram conferidas com base nos cartões de vacinação, considerando o calendário de imunização recomendado pelo Ministério da Saúde. Fatores associados à não retenção do cartão de vacinação e imunização incompleta aos 18 meses foram identificados por meio da regressão de Tobit. Cerca de 73% das mães apresentaram o cartão de vacinação do filho. A não apresentação do cartão de vacinação associou-se à idade materna < 25 anos e à participação materna em emprego remunerado. Apenas 33% e 45% das crianças tinham todas as vacinas em dia aos 12 meses e 18 meses, respectivamente. Para as vacinas com 3 doses, a taxa de atraso foi de cerca de 10% para a aplicação da 1ª dose, mas de 40% para a 3ª dose. Apesar dos atrasos, a maioria das crianças com cartão de vacinação disponível tinha cobertura acima de 90% até os 18 meses de idade. Os fatores ajustados associados à vacinação incompleta foram residir em domicílio com mais de um filho (p = 0,010) e renda mensal inferior a 1 salário mínimo (p = 0,006). Em conclusão, os atrasos na aplicação da vacina infantil foram altos durante a pandemia de COVID-19, mas houve uma adesão considerável até os 18 meses de idade. As famílias mais pobres, com mais de um filho, correm o risco de não imunizar totalmente seus filhos e devem ser alvo de políticas públicas.

## Resumen

Brasil ha experimentado una disminución en la cobertura vacunal desde el 2016. Este estudio analiza la situación vacunal de los niños nacidos durante la pandemia de COVID-19 en Fortaleza, Nordeste de Brasil. Un análisis longitudinal incluyó a 313 niños con información a los 12 y 18 meses de edad. Se revisaron los carnés de vacunación para aplicar la dosis considerando el calendario de inmunización recomendado por el Ministerio de Salud. Los factores asociados con la no retención del carné de vacunación y la inmunización incompleta a los 18 meses se identificaron mediante la regresión de Tobit. Alrededor del 73% de las madres presentaron el carné de vacunación de sus hijos. La no disponibilidad del carné de vacunación se asoció con la edad materna < 25 años y la participación materna en actividad remunerada. Solo el 33% y el 45% de los niños estaban al día con todas sus vacunas a los 12 meses y 18 meses, respectivamente. Para las vacunas de 3 dosis, la tasa de retraso fue de alrededor del 10% para la 1ª dosis, pero del 40% para la 3ª dosis. A pesar de los retrasos, la mayoría de los niños con el carné de vacunación disponible tenía una cobertura superior al 90% hasta los 18 meses de edad. Los factores ajustados asociados con la vacunación incompleta fueron vivir en un hogar con más de un hijo (p = 0,010) e ingreso mensual inferior a 1 salario mínimo (p = 0,006). En definitiva, los retrasos en la administración de la vacuna infantil fueron altos durante la pandemia de COVID-19, pero hubo una adhesión considerable hasta los 18 meses de edad. Las familias más pobres, con más de un hijo, corren el riesgo de no inmunizar completamente a sus hijos y deberían ser objeto de políticas públicas.

## Introduction

The Brazilian National Immunization Program (PNI, acronym in Portuguese) was created in 1973 and became recognized worldwide as successful due to its high vaccine coverage ^1^ ^,^ ^2^. The PNI offers vaccines free of charge to the population as part of the universal health system, helping reduce the burden of infectious diseases ^1^ ^,^ ^2^ ^,^ ^3^. However, since 2016, vaccination coverage has been decreasing in Brazil ^1^ ^,^ ^4^ ^,^ ^5^. Comparing 2015 to 2021, vaccines such as BCG and meningococcal C had near-universal coverage and decreased to about 70%, which is below the 90% to 95% target set by the Brazilian Ministry of Health for preventing outbreaks ^6^ ^,^ ^7^ ^,^ ^8^. In Fortaleza (Ceará State, Northeast Brazil), a population-based household survey conducted in 2017 found coverage rates of 99.3%, 77.3%, and 76.1% for BCG, meningococcal C, and pentavalent vaccines ^9^.

Several factors have been linked to the decrease in vaccination coverage and antivaccine movements in Brazil and the world, including the misconception that vaccines are no longer necessary to prevent diseases that have disappeared, lack of understanding of how vaccines protect from pathogens, fear of side effects and sequelae, diminished parental availability to attend to routine vaccinations of children, and concerns about a large number of immunizations in early childhood ^4^ ^,^ ^10^ ^,^ ^11^.

The COVID-19 pandemic exacerbated the decline in vaccine coverage. The pandemic affected healthcare in many ways, including disruption of services (e.g., child health services and thus access to immunization), social distancing measures that limited the search for care, delay in seeking services due to and fear of exposure to the virus ^12^ ^,^ ^13^ ^,^ ^14^, and interruption of vaccination campaigns and home visits. As a result, vaccination coverage reduced in Brazil, and many children completely lacked immunization or received vaccines with major delays, exposing them to preventable infections ^7^ ^,^ ^15^ ^,^ ^16^ ^,^ ^17^.

Despite social media’s potential to facilitate the spread of proper scientific evidenced facts about vaccination, it also likely played a role in vaccination coverage. These platforms can serve as an easy and fast mean to disseminate false information regarding vaccine safety and efficacy, especially among those who undervalue the risk of resurgence of vaccine-preventable diseases and do not check validity of information found online ^15^ ^,^ ^16^ ^,^ ^18^ ^,^ ^19^ ^,^ ^20^. Moreover, during the pandemic, the former president of Brazil spread disinformation about the COVID-19 and its vaccine, denying science and the effectiveness of vaccines. This also affected the uptake of childhood vaccines ^19^ ^,^ ^20^.

Given this context, Brazil faces a major threat due to the reintroduction of diseases previously eliminated with vaccines (e.g., poliomyelitis), and the continued expansion of diseases recently reintroduced (e.g., measles) ^12^ ^,^ ^16^ ^,^ ^21^. In this study, we leverage longitudinal data from a cohort study conducted in Fortaleza (*Iracema-COVID*) ^22^, to assess the vaccination coverage of children at 12 and 18 months of age, and to identify factors associated with delays or lack of vaccination. Children included in Iracema-COVID study were born in July and August 2020, and their mothers were pregnant during a lockdown period in the city, so both mothers and children were exposed to the negative consequences of COVID-19 on healthcare.

## Methods

This is a longitudinal analysis of the childhood vaccination coverage among children aged 12 and 18 months included in the Iracema-COVID study. The study was approved by the Research Ethics Committee of the Federal University of Ceará (protocol n. 31190420.4.0000.5054).

### Study design

The Iracema-COVID study is a longitudinal study that enrolled a sample of women who were pregnant during the lockdown period in 2020 and delivered their children from July to August 2020. The sample size was calculated based on data from the Brazilian Information System on Live Births (SINASC, acronym in Portuguese). Cohort participants consist of infants whose mothers lived in Fortaleza, delivered in public hospitals, and presented available complete address information. A total of 4,480 mothers had children during the period, and 3,567 were eligible for the study. The desired sample size was 352 mothers. To reach this number, 724 women were randomly sampled (372 additional dyads were performed in anticipation of refusals to participate and eventual problems with wrong or changed addresses) using the *GSAMPLE* module in Stata sotware (https://www.stata.com). At least three contact attempts by telephone were performed to all 724 women and, of these, 351 agreed to participate in the baseline study (later one of the mothers gave up participation), being interviewed at six months postpartum ^22^. The first survey round of the cohort was conducted from January 8, 2021 to July 18, 2021. This first round partially encompassed the second wave of the COVID-19 pandemic, so the interviews were conducted remotely, by phone, whereas the second and third rounds were conducted in person, at the participant’s home. The present study included data from two survey rounds conducted at 12 and 18 months postpartum.

### Measurements

Data on childhood vaccination were collected on three consecutive waves of the study using the question “Is your child up to date with their vaccinations?” (“yes” or “no”). Moreover, in the second (12 months) and third (18 months) survey rounds, mothers were asked for permission to photograph the vaccination card of children, and 313 records were obtained. Of these, 272 were available for evaluation in the two survey rounds, of which 242 were legible for data compilation and analysis.

To analyze possible factors associated with vaccine coverage, maternal and child characteristics were selected following a conceptual model. Maternal age was collected as complete years and grouped into two categories: 18 to 24 and ≥ 25. Schooling level was measured by the highest level of formal education reached by the mother converted into years of formal education (< 8, ≥ 8). Skin color data was collected by maternal self-reference and included white, mixed-race, or black. Marital status was also obtained and included single, married, stable union, divorced, or widowed; however, they were grouped as married or single. Parity considers the number of children. Working mother considers whether the mother has a job. Mothers’ report on participation in any government cash transfer program was collected. Monthly family income was based on mothers’ report, latter on categorized into Brazilian minimum wages. Maternal and children morbidity considered any episode of illness in the six months prior to the interview. Maternal depressive symptoms were assessed by the *Self-Report Questionnaire* (SRQ-20), a 20-item self-report screening tool developed by the World Health Organization (WHO) to detect psychological distress ^23^.

Based on the review of the child vaccination cards, an individual variable for each vaccine was created, composed of three categories: “up to date vaccination”, “delayed vaccination”, and “no vaccination”, following the vaccination schedule proposed by the Brazilian Ministry of Health ^24^. With these variables, “Child’s immunization status” was obtained, which shows the number of vaccines the child was fully vaccinated for by 18 months of age.

### Data analysis

Descriptive statistics of maternal and child characteristics were obtained at cohort wave. For the univariate analysis, 95% confidence intervals (95%CI) were estimated, whereas for the bivariate analysis, associations between outcomes and their covariates were assessed by chi-square tests.

The agreement between the mothers’ self-reporting of the up-to-date child vaccination and the card checking of the up-to-date child vaccination schedule, at 12 months and 18 months, was analyzed based on kappa’s concordance test.

The analyzed outcome was the number of vaccines with complete number of due shots taken, for each child from birth to 18 months. For this censored outcome variable that ranged from two to 12 vaccines, a Tobit regression model was used to estimate the mean change in the response variable “number of completed vaccines” for units in the covariates. In situations in which the outcome variable was censored, ordinary least squares (OLS) regression, which is commonly used for continuous variables, was considered inappropriate. Tobit regression is specifically designed to handle censored data by accounting for both the observed and unobserved portions of the outcome variable. For each predictor variable, the slope coefficient (β) and their respective 95%CI were reported for the associations between children’s vaccine number and the investigated variables. Statistical analysis was performed in Stata, version 16.1.

## Results

The vaccination status of 325 children born during the COVID-19 pandemic was assessed in their first 18 months of life. Children had a similar distribution by sex, 9.54% were low birth weighed, and 61,46% reported some illness in the previous six months. Regarding primary health care consultation during the first 18 months. Their mothers’ profile showed that 29.85% of mothers were young (aged 18 to 24 years old), 9.85% had only elementary schooling, 44.9% had a job, and 44% received government cash transfers. Concerning their health status, 10.15% reported an illness in the previous six months and 25.85% were identified with common mental disorder. Of the 325 mothers, 313 (96.3%) had their children’s vaccination cards available. From these, 31 (9.9%) showed the cards only at the 12 months after birth, 31 (9.9%) only at 18 months, and 229 (73.16%) at both moments (Table 1).

**Table 1:** Maternal and child characteristics at baseline of the Iracema-COVID study. Fortaleza, Ceará State, Brazil, 2020-2022.

Characteristics	n	%	95%CI
Maternal			
Age (years)			
< 25	97	29.85	25.10; 35.06
≥ 25	228	70.15	64.93; 74.89
Skin color			
White	59	18.15	14.31; 22.74
Mixed-race	228	70.15	64.93; 74.89
Black	35	10.77	7.82; 14.64
Marital status			
Single	229	70.46	65.28; 75.16
Married	96	29.54	24.81; 34.74
Schooling level (years)			
< 8	32	9.85	7.03; 13.60
≥ 8	293	90.15	86.39; 92.96
Parity			
Primiparous	151	68.02	61.56; 73.85
Multiparous	71	31.98	26.14; 38.43
Working mother			
Yes	141	44.90	39.46; 50.46
No	173	55.10	49.53; 60.53
Cash transfer program			
Yes	143	44.00	38.67; 49.46
No	182	56.00	50.53; 61.32
Monthly family income (minimum wage)			
< 1	48	14.77	11.30; 19.07
1-2	183	56.31	50.84; 61.62
3 or above	94	28.92	24.23; 34.10
Morbidity in the previous six months			
Yes	33	10.15	7.29; 13.95
No	292	89.85	86.04; 92.70
SRQ-20 *			
< 8	241	74.15	69.12; 78.62
≥ 8	84	25.85	21.38; 30.88
Pattern of maternal consultation in the three waves			
Full attendance	93	29.62	24.81; 34.92
Start attending	50	15.92	12.26; 20.41
Stop attending	89	28.34	23.61; 33.60
No attendance	82	26.11	21.54; 31.27
Child			
Sex			
Male	167	51.38	45.93; 56,80
Female	158	48.62	43.19; 54.06
Low birth weight			
Yes	31	9.54	6.77; 13.26
No	294	90.46	86.73; 93.22
Morbidity in the previous six months			
Yes	193	61.46	55.94; 66.70
No	121	38.54	33.29; 44.05
Child consultation during the three waves			
Full attendance	234	74.52	69.39; 79.05
Start attending	16	5.10	3.13; 8.16
Stop attending	52	16.56	12.83; 21.10
No attendance	12	3.82	2.17; 6.62
Availability of vaccination card			
None	22	7.03	4.66; 10.45
By 12 months	31	9.90	7.04; 13.75
By 18 months	31	9.90	7.04; 13.75
By 12 and 18 months	229	73.16	67.95; 77.79

95%CI: 95% confidence interval; SRQ-20: *Self-Reporting Questionnaire*.

* < 8: negative, ≥ 8: positive.

Factors associated with having a vaccination card were maternal age and mother’s employment status. Mothers aged < 25 years had a greater tendency not to show the vaccination card (42.86%) compared to those who did (26.2%) in both waves, and almost half of the formally employed mothers presented the vaccination card in both moments of the survey (41.48%) (Table 2).

**Table 2:** Availability of the child’s vaccination card at 12 and 18 months of life, according to maternal and child characteristics. Iracema-COVID study, Fortaleza, Ceará State, Brazil, 2020-2022.

Characteristics	Did not present vaccine card in at least one follow-up round		Presented vaccine card in both follow-up rounds		p-value
	n	%	n	%	
Maternal					
Age (years)					0.005
< 25	36	42.86	60	26.20	
≥ 25	48	57.14	169	73.80	
Skin color					0.367
White	19	22.62	38	16.59	
Mixed-race	58	69.05	160	69.87	
Black	7	8.33	28	12.23	
Others	0	0.00	3	1.31	
Marital status					0.231
Single	50	59.52	153	66.81	
Married/Stable union	34	40.48	76	33.19	
Schooling (years)					0.862
< 8	9	10.71	23	10.04	
≥ 8	75	89.29	206	89.96	
Parity					0.409
Primiparous	40	63.49	106	69.28	
Multiparous	23	36.51	47	30.72	
Working mother					0.036
Yes	46	54.76	95	41.48	
No	38	45.24	134	58.52	
Cash transfer program					0.418
Yes	43	51.19	129	56.30	
No	41	48.81	100	43.67	
Monthly family income (minimum wage)					0.486
< 1	26	30.95	56	24.45	
1	42	50.00	129	56.33	
2 or more	16	19.05	44	19.21	
SRQ-20					0.372
< 8	64	76.19	185	80.79	
≥ 8	20	23.81	44	19.21	
Maternal consultation in third wave					0.577
Yes	33	39.29	98	42.79	
No	51	60.71	131	57.21	
Child					
Sex					0.055
Male	51	60.71	111	48.40	
Female	33	39.29	118	51.53	
Low birth weight					0.649
Yes	77	91.67	206	89.96	
No	7	8.33	23	10.04	

SRQ-20: *Self-Reporting Questionnaire*.

* < 8: negative, ≥ 8: positive.

When analyzing the adequacy of the moment in which the vaccines were taken (n = 242), having as a reference what the Brazilian Ministry of Health recommends, and allowing a tolerance margin of two months, it was observed, among mothers who presented the vaccination card, that only 32.5% and 44.6% of the children had all ten vaccines up to date at 12 and 18 months, respectively. On the other hand, 47.4% of the children had one to five vaccines overdue at 12 months, whereas 43.2% remained overdue at 18 months. In contrast, by analyzing the children with vaccination self-reported by their mothers, 43.9% were overdue for one to five vaccines at 12 months and 51.6% at 18 months in the period analyzed. Regarding vaccination delays, data from vaccination cards broadly matched mothers’ self-reporting. Applied kappa test showed concordance of 70.5% (kappa = -0.0073; p = 0.550) by 12 months and 59.2% (kappa = -0.0423; p = 0.800) at 18 months (Table 3).

**Table 3:** Vaccination pattern * of children at 12 and 18 months of age, according to the vaccination card and the mother’s self-report. Iracema-COVID study, Fortaleza, Ceará State, Brazil, 2020-2022.

	Delayed vaccination **					
	By 12 months			By 18 months		
	No vaccine	1-5 vaccines	5-10 vaccines	No vaccine	1-5 vaccines	5-10 vaccines
	n (%)	n (%)	n (%)	n (%)	n (%)	n (%)
Mothers who showed *** a vaccination card						
Vaccine card ^#^	37 (32.5)	54 (47.4)	23 (20.2)	66 (44.6)	64 (43.2)	18 (12.2)
Self-report ^#^	30 (30.6)	43 (43.9)	25 (25.5)	28 (30.8)	47 (51.6)	16 (17.6)
Mothers who did not show *** a vaccination card						
Self-report	5 (62.5)	1 (12.5)	2 (25)	4 (57.1)	2 (28.6)	1 (14.3)

* Considering the 10 following vaccines: BCG, hepatitis B, rotavirus, Haemophilus influenza type B, pentavalent, polio VIP, polio VOP, triple viral, DTP, and hepatitis A;

** For mothers who presented vaccination card, overdue was considered when vaccines were applied after two months of the scheduled date; for those without card, the vaccine delay was self-reported by the mothers;

*** Mothers who presented a vaccination card only at 12 (n = 114) or 18 months (n = 148); mothers who did not present a vaccination card: at 12 (n = 57) and 18 months (n = 61);

^#^ Kappa test of agreement between vaccine cards and mothers’ self-report: at 12 months, agreement of 70.46% was observed (kappa = -0.0073; p = 0.55), whereas agreement of 59.21% was observed at 18 months (kappa = -0.0423; p = 0.80).

Table 4 shows that, based on the vaccination cards, BCG, and hepatitis B vaccines, usually given in hospitals at birth, were applied in 97,5% of the children. However, vaccines that are given at health facilities after birth had lower coverage: 9,5% of children did not receive the first dose of polio VIP at two months of age, and 20% did not receive the triple viral dose at 12 months of age. Delays in vaccination were also common. The third dose of the polio VIP vaccine was delayed in more than half of the children, compared with 12,2% in the first and 23,8% in the second dose.

**Table 4:** Pattern of vaccine application at 18 months of age, according to the vaccination schedule. Iracema-COVID study, Fortaleza, Ceará State, Brazil, 2020-2022.

Vaccines	Expected date	Not applied	Applied in the appropriate period *	Applied with delay **	Delay in days
		n (%)	n (%)	n (%)	Median (SD)
BCG	At birth	2 (0.7)	281 (95.9)	10 (3.4)	2 (57.0)
Hepatitis B	At birth	5 (1.8)	277 (94.5)	11 (3.7)	2 (81.2)
Pentavalent					
Dose 1	2 months	2 (0.7)	259 (87.2)	36 (12.1)	88 (16.0)
Dose 2	4 months	3 (1.0)	223 (75.1)	71 (23.9)	36 (58.8)
Dose 3	6 months	12 (4.1)	134 (46.0)	145 (49,9)	27 (58.5)
Polio VIP					
Dose 1	2 months	28 (9.5)	231 (78.3)	36 (12.2)	65 (28.8)
Dose 2	4 months	3 (1.0)	221 (75.2)	70 (23.8)	36 (55.0)
Dose 3	6 months	10 (3.5)	132 (45.8)	146 (50.7)	26 (50.4)
Polio VOP					
Booster 1	15 months	76 (25.6)	123 (41.4)	98 (33.0)	53 (51.6)
Booster 2	4 years	-	-	-	
Pneumococcal 10					
Dose 1	2 months	4 (1.3)	263 (88.3)	31 (10.4)	71 (26.2)
Dose 2	4 months	6 (2.0)	224 (75.4)	67 (22.6)	34 (41.8)
Rotavirus					
Dose 1	2 months	8 (2.7)	259 (87.5)	29 (9.8)	33 (35.5)
Dose 2	4 months	13 (4.4)	220 (74.6)	62 (21.0)	32 (21.0)
Meningococcal					
Dose 1	3 months	6 (2.0)	232 (78.1)	59 (19.9)	34 (58.6)
Dose 2	5 months	12 (4.1)	179 (61.1)	102 (34.8)	34 (49.5)
Dose 3	12 months	38 (12.8)	136 (46.0)	122 (41.2)	48 (48.8)
Yellow fever					
Dose 1	9 months	217 (73.3)	3 (1.0)	76 (25.7)	228 (56.7)
Booster	4 years	-	-	-	-
Triple viral					
Unique dose	12 months	47 (20.0)	77 (32.9)	110 (47.1)	50 (41.1)
DTP					
Dose 1	15 months	77 (25.9)	122 (41.1)	98 (33.0)	48 (52.7)
Booster	4 years	-	-	-	
Hepatitis A					
Unique dose	15 months	86 (29.0)	119 (40.2)	91 (30.8)	49 (52.7)

SD: standard deviation.

* Application of the vaccine dose at the appropriate time or up to two months late;

** Application of the vaccine dose with two or more months of delay from the expected date.

Despite delays in doses application, by 18 months of age most children with available vaccination card were fully immunized with the main vaccines recommended by the Brazilian Ministry of Health. Vaccines with more than 90% of children with available vaccination card with fully immunized status were: BCG (98.35%), hepatitis B (97.12%), Haemophilus influenza type B (98.35%), rotavirus (95.04%), pentavalent (94.63%), and polio VIP (93.8%). However, considering complete vaccination, we highlight that, for vaccines of strategic importance such as triple viral and oral polio vaccine, about one fourth of the children had an incomplete schedule for their age. Hepatitis A (69.01%) and yellow fever (25.21%) also presented very low proportion of fully immunized children (Table 5). Nevertheless, it is worth mentioning that these results do not refer to all children, but to those with vaccination cards available for assessment.

**Table 5:** Complete immunization among children with available vaccination card at 18 months of age, according to type and number of vaccines. Iracema-COVID study, Fortaleza, Ceará State, Brazil, 2021-2022.

	Fully immunized	
	n	%
Vaccines		
BCG (1 dose)	238	98.35
Hepatitis B (1 dose)	235	97.12
Haemophilus influenza type B (2 doses)	238	98.35
Rotavirus (2 doses)	230	95.04
Pentavalent (3 doses)	229	94.63
Polio VIP (3 doses)	227	93.80
Meningococcal C (3 doses)	209	86.36
Triple viral (1 dose)	187	77.27
VOP (3 doses)	178	73.55
Hepatitis A (1 dose)	167	69.01
Yellow fever (1 dose)	61	25.21
Number of complete vaccines per child with vaccination card		
12	42	17.28
11	94	38.68
10	20	8.23
9	18	7.41
8	25	10.29
7	9	3.70
6	24	9.88
5	6	2.47
4	3	1.23
2	2	0.82

We created a variable with the number of complete vaccines per child, with values ranging from 1 to 12. This discrete numeric outcome was related to the predictor variable to identify factors associated with child full immunization. Table 6 shows that vaccine completeness is positively associated with older maternal age (p = 0.014) and with higher monthly family income (p = 0.008), but negatively associated with having more than one sibling aged below six years (p = 0.039) and children above six years old living at home (p = 0.002). Therefore, results suggest that an increasing number of siblings reduce the number of complete vaccines.

**Table 6:** Final model of Tobit regression with the adjusted factors * associated with the outcome “number of complete vaccines” **. Iracema-COVID study, Fortaleza, Ceará State, Brazil, 2021-2022.

Characteristics	β (95%CI)	p-value
Maternal		
Age (years)		
< 25	1.00	1.000
≥ 25	0.06 (0.012; 0.11)	0.014
Children under 6 years		
1	1.00	1.000
2	-0.84 (-1.63; -0.04)	0.039
3	1.51 (-0.97; 4.01)	0.231
5 or more	12.49 (-930.50; 955.48)	0.979
Children above 6 years		
None	1.00	1.000
1	-0.71 (-1.45; 0.01)	0.056
2	-1.46 (-2.49; -0.43)	0.006
3	-3.64 (-5.90; -1.37)	0.002
4	-3.49 (-8.36; 1.37)	0.158
5 or more	-0.92 (-5.73; 3.89)	0.707
Monthly income (minimum wage)		
< 1	1.00	1.000
1	1.06 (0.28; 1.85)	0.008
2 or more	0.98 (-0.007; 1.97)	0.052

95%CI: 95% confidence interval.

* Adjusted by the above variables and mother’s skin color, maternal working, cash transfer program participation, maternal morbidity, maternal depression, previous mother’s primary health care consultation, child sex, low birth weight, and child morbidity;

** Complete immunizations among children 18 months of age with available vaccination card.

## Discussion

The present cohort study analyzed the vaccination status of 325 children born during the COVID-19 pandemic in Fortaleza, the region with the highest poverty rates in the country ^24^. Of these children, 313 had the child’s vaccination card available (96.3%), but only 229 (73.2%) mothers showed their child’s vaccination card at both follow-up rounds. Of the children with vaccination cards, only 33% and 45% had all vaccines up to date, at 12 months and 18 months, respectively. For 3-dose vaccines, the delay rate was around 10% for the first dose application but 40% for the third dose. Despite delays, by 18 months of age, most children with available vaccine cards had completed the vaccination schedule: above 95% were fully immunized with BCG, hepatitis B, rotavirus, pneumococcal 10, polio VIP, and pentavalent. However, less than 80% were immunized with triple viral, polio VOP, hepatitis A, and yellow fever. Adjusted factors associated with incomplete vaccination were living in a household with more than one child (p = 0.010) and monthly income of less than one minimum wage (p = 0.006).

Retention of the vaccine card has been analyzed in other studies. For instance, a rate of 78% for children aged one to three years was found in Buenos Aires (Argentina) ^25^. Moreover, among low- and middle-income countries, having a vaccine card ranged from 69% in South Africa to just 21% in the Democratic Republic of the Congo ^26^.

Although vaccination providers are advised against refusing vaccination to those without a card ^26^, not showing a vaccination card at the time of visiting a clinic can impair the assessment of the child’s vaccination status, leading to the loss of the opportunity to vaccinate ^27^ ^,^ ^28^ ^,^ ^29^. In this study, factors associated with the non-availability of vaccination cards were maternal age below 25 years and the mother having a paid job. A study recently conducted in Yaoundé (Cameroon) corroborate our results ^30^. It suggests that mothers who are employed have little time to follow their children’s immunization and might occasionally delegate this to a third party (say a family member), with vaccination card getting missed consequently ^30^. Another study, conducted in Karachi (Pakistan), identified overcrowding in the household and children older than four years of age as factors associated with not retaining the vaccination card ^31^.

A distinct pattern was found in southern Italy, where 78% of children were adequately vaccinated for their ages, 6% were overdue, and 16% had not received any dose of any vaccine ^32^. This total absence of application of any vaccine dose suggests a strong anti-vaccine attitude prevailing in part of the population, perhaps influenced by no-vax movements. This phenomenon was not observed in the present study since all children received some doses of various vaccines, albeit inadequately.

Our results show that, in addition to checking the vaccination card, mothers were also asked if they considered that their children’s vaccinations were up to date. There was an important agreement between what the card showed and what the mother reported, both at 12 and 18 months, showing that reports were quite reliable, which can be useful in cases requiring quick diagnosis.

Studies that performed a similar comparison in Senegal and Ethiopia also found strong agreement between vaccine registration documents and maternal recall ^33^ ^,^ ^34^ ^,^ ^35^. In the Senegalese study, however, a large discrepancy was observed between the vaccine card and the mother’s recall for the polio vaccine, suggesting that this may be because mothers only considered vaccines given via injection ^33^.

When the delay was analyzed for each vaccine, we found a clear pattern of increasing delay from dose to dose for vaccines that require more than one application. For vaccines that require three doses, the delay rate increased from 10% to 12% in the application of the first dose and from 41% to 51% in the third dose.

Other studies observed a similar pattern. In Nepal, for instance, age-appropriate pentavalent coverage dropped from 74% at the first dose to 42% at the third dose ^36^; in China, age-appropriate coverage for measles decreased from 77% to 68% from the first to the second dose, respectively ^37^.

Despite delays in administering doses, it was observed that most children aged 18 months with vaccination cards had completed the vaccination schedule: more than 95% had received a single dose of BCG and hepatitis B at birth, both doses of rotavirus and pneumococcal 10 and three doses of polio VIP and pentavalent. Negatively, we highlight that about one fourth of the children had an incomplete schedule for their age for vaccines of strategic importance such as triple viral and oral polio vaccines. This is worrisome since both measles and polio are diseases with persistence and reintroduction potential, respectively, in our country. Although vaccines against hepatitis A and yellow fever are part of the children’s schedule throughout the Brazilian national territory, the proportions of children fully immunized against these diseases were considerably low. However, we highlight that these results do not refer to all children, but to those with vaccination cards available for assessment.

In 2012, the World Health Assembly called on all countries to reach ≥ 90% national coverage with all vaccines in the immunization schedule by 2020 ^38^. It seems the goal was only partially achieved ^39^, as a considerable proportion of study children with vaccination cards were not fully immunized by all scheduled vaccines at 18 months of age. Data from Nepal shows that the country has achieved the goal of above 90% coverage for all vaccines ^35^. A similar pattern was observed in studies conducted in Ghana, where 89% of the children aged 12-23 months were fully immunized for the recommended seven vaccines ^28^ ^,^ ^29^.

For children of the same age group in one state in Nigeria, the full immunization rate reached 79% ^40^. Senegal achieved 71% of the 12 to 36 months old children fully immunized ^41^, whereas vaccination coverage was only 62% in India, despite presenting a 19% increase in one decade ^42^. European countries had high rates of full immunization for decades, reaching around 95% overall, but that has lately been declining, with some countries experiencing unprecedented outbreaks of vaccine-preventable diseases ^43^.

When considering the number of complete vaccines of each child at 18 months of age as response variable, adjusted linear regression analysis found the following factors associated with incomplete vaccination: having more than one child (p = 0.010) and belonging to a family with a monthly income of less than one minimum wage (p = 0.006). Families receiving the Brazilian Income Transfer Program (*Bolsa Família* program) were more likely to have incomplete vaccination. A longitudinal study conducted with 3,242 families of *Bolsa Família* program found the highest proportion of adequate vaccination among children aged 12 months belonging to the richest quintile (67.9%) and among children whose mothers had ≥ 9 years of schooling (63.3%). At 18 months, no differences were observed between the proportions of adequate vaccination according to income or schooling ^44^. When launched, *Bolsa Família* was a conditional cash transfer, and one of the conditionalities was updated vaccination of children. However, enforcement and monitoring of those conditionalities have become more flexible ^45^ ^,^ ^46^.

Predictors similar to those found in our study were also identified in studies conducted in Nigeria and in a Chinese province, which found incomplete immunization associated with low-income families ^37^ ^,^ ^40^, and also with families with more than one child, as found in studies conducted in Ghana and China ^28^ ^,^ ^37^. Other predictors of full immunization included high maternal education, urban settings, higher number of antenatal care consultations, and mothers with no fixed job, in countries such as Senegal and China ^37^ ^,^ ^41^. A study carried out in Buenos Aires, however, found that high maternal schooling level showed a strong significant association with hesitancy to vaccination ^25^.

The main strength of this paper is the longitudinal pattern of vaccination of children during the COVID-19 pandemic, while most recent vaccination studies have focused on specific questions related to the COVID-19 vaccine. We analyzed not only the issue of vaccination coverage, but also the retention of the vaccination document over time, the delay in the application of doses of each vaccine, and the achievement of complete immunization at 18 months of life. In addition, we were able to identify factors associated with the loss of vaccination cards and with non-complete vaccination in the context of social distancing due to the COVID-19 pandemic.

This study presents some limitations. Our results are representative of mothers with infants born during the COVID-19 pandemic in public hospitals in the fifth largest city in Brazil. Thus, they are representative of large urban areas in the country and should not be generalized to other contexts. The outcomes were based on the review of vaccination cards made available to us by the mothers. Thus, although recall bias was not a concern, some mothers (7%) did not show the cards. No sample calculation was performed for possible associations with other outcomes, in addition to the calculation for the main outcome of the study (common mental disorder), which might have an impact on its sampling power. Moreover, the sample was not increased in size for confounding control purposes. There were 10% losses due to follow-up, and no analysis was performed to assess possible attrition bias. Additionally, the interaction with the interviewers and the recurrent assessment of the children’s vaccination status may have encouraged families to vaccinate their infants. We were not able to verify this possible interference since there was not a control group of mothers.

The longitudinal study design aimed to characterize patterns of child vaccination during the first 18 months of life. This provides a comprehensive description of the mother’s child vaccination behavior along several phases of restrictive measures of the COVID-19 pandemic. We found that only a small proportion of mothers (7%) did not retain the child vaccination card for up to 18 months. Although two-thirds of the mothers had delayed the child vaccination schedule at 12 months of age, an approximation was observed by 18 months, in which 95% of children were fully immunized for some of the main vaccine-preventable diseases. Mothers with more than one child and living in a low-income family (below one minimum wage) were particularly at risk of a child not fully immunized and should be the target of public policies.

## References

[1] Domingues CMAS, Teixeira AMDS (2013). Coberturas vacinais e doenças imunopreveníveis no Brasil no período 1982-2012: avanços e desafios do Programa Nacional de Imunizações.. Epidemiol Serv Saúde.

[2] Domingues CMAS, Maranhão AGK, Teixeira AM, Fantinato FFS, Domingues RAS (2020). The Brazilian National Immunization Program 46 years of achievements and challenges. Cad Saúde Pública.

[3] Silva JBD (2013). 40 years do Programa Nacional de Imunizações uma conquista da saúde pública brasileira. Epidemiol Serv Saúde.

[4] Olive JK, Hotez PJ, Damania A, Nolan MS (2018). The state of the antivaccine movement in the United States a focused examination of nonmedical exemptions in states and counties. PLoS Med.

[5] Conselho Nacional das Secretarias de Saúde A queda da imunização no Brasil..

[6] Fundação Maria Cecília Souto Vidigal Desigualdades e impactos da COVID-19 na atenção à primeira infância..

[7] Sato APS (2020). Pandemic and vaccine coverage challenges of returning to schools. Rev Saúde Pública.

[8] Césare N, Mota TF, Lopes FFL, Lima ACM, Luzardo R, Quintanilha LF (2020). Longitudinal profiling of the vaccination coverage in Brazil reveals a recent change in the patterns hallmarked by differential reduction across regions. Int J Infect Dis.

[9] Maciel JAP, Silva AC, Campos JS, Correia LL, Rocha HAL, Rocha SGMO (2019). Análise do estado de cobertura vacinal de crianças menores de três anos no município de Fortaleza em 2017. Rev Bras Med Fam Comunidade.

[10] Brown AL, Sperandio M, Turssi CP, Leite RMA, Berton VF, Succi RM (2018). Vaccine confidence and hesitancy in Brazil. Cad Saúde Pública.

[11] Oliveira IS, Cardoso LS, Ferreira IG, Alexandre-Silva GM, Jacob BCS, Cerni FA (2022). Anti-vaccination movements in the world and in Brazil. Rev Soc Bras Med Trop.

[12] Dinleyici EC, Borrow R, Safadi MAP, Van Damme P, Munoz FM (2021). Vaccines and routine immunization strategies during the COVID-19 pandemic. Hum Vaccin Immunother.

[13] Ota MOC, Badur S, Romano-Mazzotti L, Friedland LR (2021). Impact of COVID-19 pandemic on routine immunization. Ann Med.

[14] Lassi ZS, Naseem R, Salam RA, Siddiqui F, Das JK (2021). The impact of the COVID-19 pandemic on immunization campaigns and programs a systematic review. Int J Environ Res Public Health.

[15] Wilson SL, Wiysonge C (2020). Social media and vaccine hesitancy. BMJ Glob Health.

[16] Freeman RE, Thaker J, Daley MF, Glanz JM, Newcomer SR (2022). Vaccine timeliness and prevalence of undervaccination patterns in children ages 0-19 months, U S., National Immunization Survey-Child 2017. Vaccine.

[17] Nelson R (2020). COVID-19 disrupts vaccine delivery. Lancet Infect Dis.

[18] Al-Uqdah L, Franklin FA, Chiu CC, Boyd BN (2022). Associations between social media engagement and vaccine hesitancy. J Community Health.

[19] Phadke VK, Bednarczyk RA, Salmon DA, Omer SB (2016). Association between vaccine refusal and vaccine-preventable diseases in the United States. JAMA.

[20] Dubé E, Laberge C, Guay M, Bramadat P, Roy R, Bettinger JA (2013). Vaccine hesitancy. Hum Vaccin Immunother.

[21] Westerman R, Dorea F, Hussein IH, Raslan R, Sayegh SE, Chams S (2017). Re-emerging vaccine-preventable diseases in war-affected peoples of the Eastern Mediterranean Region an update. Front Public Health.

[22] Castro MC, Farías-Antúnez S, Araújo DABS, Penna AL, Oliveira FA, Aquino CM (2022). Cohort profile maternal and child health and parenting practices during the COVID-19 pandemic in Ceará, Brazil: birth cohort study (Iracema-COVID). BMJ Open.

[23] Mari J, Williams P (1986). A validity study of a psychiatric screening questionnaire (SRQ-20) in primary care in the city of Sao Paulo. Br J Psychiatry.

[24] Silva JJ, Bruno MAP, Silva DBN (2020). Pobreza multidimensional no Brasil uma análise do período 2004-2015. Brazilian Journal of Political Economy.

[25] Gentile A, Pacchiotti AC, Giglio N, Nolte MF, Talamona N, Rogers V (2021). Vaccine hesitancy in Argentina validation of WHO scale for parents. Vaccine.

[26] Wagner AL (2019). The use and significance of vaccination cards. Hum Vaccin Immunother.

[27] Rainey JJ, Watkins M, Ryman TK, Sandhu P, Bo A, Banerjee K (2011). Reasons related to non-vaccination and under-vaccination of children in low and middle income countries findings from a systematic review of the published literature, 1999-2009. Vaccine.

[28] Adokiya MN, Baguune B, Ndago JA (2017). Evaluation of immunization coverage and its associated factors among children 12-23 months of age in Techiman Municipality, Ghana, 2016. Arch Public Health.

[29] Baguune B, Ndago JA, Adokiya MN (2017). Immunization dropout rate and data quality among children 12-23 months of age in Ghana. Arch Public Health.

[30] Yakum MN, Funwie AD, Ajong AB, Tsafack M, Ze LEE, Ekukole ESR (2022). Factors associated with routine vaccination card retention among children aged 0-59 months in Yaounde-Cameroon a cross-sectional survey. PLoS One.

[31] Sheikh SS, Ali SA (2014). Predictors of vaccination card retention in children 12-59 months old in Karachi, Pakistan. Oman Med J.

[32] Lo Vecchio A, Cambriglia MD, Fedele MC, Basile FW, Chiatto F, Miraglia del Giudice M (2019). Determinants of low measles vaccination coverage in children living in an endemic area.. Eur J Pediatr.

[33] Seror V, Cortaredona S, Ly EY, Ndiaye S, Gaye I, Fall M (2020). Vaccination card availability and childhood immunization in Senegal. BMC Public Health.

[34] Porth JM, Wagner AL, Tefera YA, Boulton ML (2019). Childhood immunization in Ethiopia accuracy of maternal recall compared to vaccination cards. Vaccines (Basel).

[35] Miles M, Ryman TK, Dietz V, Zell E, Luman ET (2013). Validity of vaccination cards and parental recall to estimate vaccination coverage a systematic review of the literature. Vaccine.

[36] Rauniyar SK, Iwaki Y, Yoneoka D, Hashizume M, Nomura S (2021). Age-appropriate vaccination coverage and its determinants in children aged 12-36 months in Nepal a national and subnational assessment. BMC Public Health.

[37] Hu Y, Wang Y, Chen Y, Liang H, Chen Z (2018). Measles vaccination coverage, determinants of delayed vaccination and reasons for non-vaccination among children aged 24-35 months in Zhejiang province, China. BMC Public Health.

[38] World Health Organization (2013). Global vaccine action plan 2011-2020.

[39] Strategic Advisory Group of Experts on Immunization, World Health Organization The Global Vaccine Action Plan 2011-2020. Review and lessons learned..

[40] Eze P, Agu UJ, Aniebo CL, Agu SA, Lawani LO, Acharya Y (2021). Factors associated with incomplete immunisation in children aged 12-23 months at subnational level, Nigeria a cross-sectional study. BMJ Open.

[41] Sarker AR, Akram R, Ali N, Chowdhury ZI, Sultana M (2019). Coverage and determinants of full immunization vaccination coverage among Senegalese children. Medicina (Kaunas).

[42] Priya PK, Pathak VK, Giri AK (2020). Vaccination coverage and vaccine hesitancy among vulnerable population of India. Hum Vaccin Immunother.

[43] Bechini A, Boccalini S, Ninci A, Zanobini P, Sartor G, Bonaccorsi G (2019). Childhood vaccination coverage in Europe impact of different public health policies. Expert Rev Vaccines.

[44] Barcelos RS, Santos I, Munhoz TN, Blumenberg C, Bortolotto CC, Matijasevich A (2021). Vaccination coverage in children up to 2 years old, receiving financial support from the Family Income Transfer Program, Brazil.. Epidemiol Serv Saúde.

[45] Carvalho AT, Almeida ER, Jaime PC (2014). Condicionalidades em saúde do programa Bolsa Família - Brasil uma análise a partir de profissionais da saúde. Saúde Soc.

[46] Silva FS, Queiroz RCS, Branco MRFC, Simões VMF, Barbosa YC, Rodrigues MAFR (2020). Programa Bolsa Família e vacinação infantil incompleta em duas coortes brasileiras. Rev Saúde Pública.

